# Racial Disparities in Opioid Analgesia Administration Among Adult Emergency Department Patients with Abdominal Pain

**DOI:** 10.5811/westjem.2022.8.55750

**Published:** 2022-10-24

**Authors:** Angela F. Jarman, Alexander C. Hwang, Julia P. Schleimer, Roderick W. Fontenette, Bryn E. Mumma

**Affiliations:** * University of California, Davis School of Medicine, Department of Emergency Medicine, Davis, California; † David Grant Medical Center, Travis Air Force Base, Fairfield, California; ‡ University of California, Davis School of Medicine, Violence Prevention Research Program, Department of Emergency Medicine, Davis, California; § University of California, Davis, University of California Firearm Violence Research Center, Davis, California

## Abstract

**Introduction:**

Racial disparities in pain management have been reported among emergency department (ED) patients. In this study we evaluated the association between patients’ self-identified race/ethnicity and the administration of opioid analgesia among ED patients with abdominal pain, the most common chief complaint for ED presentations in the United States.

**Methods:**

This was a retrospective cohort study of adult (age ≥18 years) patients who presented to the ED of a single center with abdominal pain from January 1, 2019–December 31, 2020. We collected demographic and clinical information, including patients’ race and ethnicity, from the electronic health record. The primary outcome was the ED administration of any opioid analgesic (binary). Secondary outcomes included the administration of non-opioid analgesia (binary) and administration of any analgesia (binary). We used logistic regression models to estimate odds ratios (OR) of the association between a patient’s race/ethnicity and analgesia administration. Covariates included age, sex, initial pain score, Emergency Severity Index, and ED visits in the prior 30 days. Subgroup analyses were performed in non-pregnant patients, those who underwent any imaging study, were admitted to the hospital, and who underwent surgery within 24 hours of ED arrival.

**Results:**

We studied 7,367 patients: 45% (3,314) were non-Hispanic (NH) White; 28% (2,092) were Hispanic/Latinx; 19% (1,384) were NH Black, and 8% (577) were Asian. Overall, 44% (3,207) of patients received opioid analgesia. In multivariable regression models, non-White patients were less likely to receive opioid analgesia compared with White patients (OR 0.73, 95% CI 0.65–0.83 for Hispanic/Latinx patients; OR 0.62, 95% CI 0.54–0.72 for Black patients; and OR 0.64, 95% CI 0.52–0.78 for Asian patients). Black patients were also less likely to receive non-opioid analgesia, and Black and Hispanic/Latinx patients were less likely than White patients to receive any analgesia. The associations were similar across subgroups; however, the association was attenuated among patients who underwent surgery within 24 hours of ED arrival.

**Conclusion:**

Hispanic/Latinx, Black, and Asian patients were significantly less likely to receive opioid analgesia than White patients when presenting to the ED with abdominal pain. Black patients were also less likely than White patients to receive non-opioid analgesia.

## INTRODUCTION

Abdominal pain is the most common presenting complaint in emergency departments (ED) in the United States, accounting for 12% of all visits.^1^,^2^ Despite historical precedent for withholding opioid analgesia in abdominal pain, opioids are currently considered standard of care in moderate and severe acute abdominal pain.^3^,^4^ Opioid analgesia decreases pain without affecting diagnostic accuracy and improves patient satisfaction.^5^–^8^ Most ED patients with abdominal pain receive analgesia, although the type, amount, and route are not standardized.^9^

Racial disparities in the treatment of pain have been found across the continuum of healthcare,^10^ from the prehospital setting^11^,^12^ to postoperative care.^13^ While data on analgesia administration in the ED is mixed, racial disparities have been reported for several painful conditions.^14^ A recent meta-analysis, which included studies of analgesia in EDs for long-bone fracture, back pain, musculoskeletal pain, and trauma, found that non-White patients were less likely than White patients to receive any analgesia and specifically opioid analgesia.^15^

The limited available evidence suggests that racial disparities extend to the management of acute, undifferentiated abdominal pain in the adult ED population. However, this data is greater than 10 years old and often does not consider ethnicity or differentiate between non-White racial groups.^16^,^17^ To evaluate whether this disparity persists in the contemporary ED and whether it affects patients of other non-White race/ethnicities, we evaluated opioid analgesia use for non-Hispanic (NH) White, Hispanic/Latinx, NH Black, and Asian patients who presented to the ED with abdominal pain. We hypothesized that disparities previously demonstrated in abdominal pain and other painful conditions would persist among the adult ED population with acute abdominal pain and extend to patients of other minoritized races/ethnicities.

## METHODS

### Study Setting and Design

We conducted a retrospective cohort study from January 1, 2019–December 31, 2020, at a single academic, tertiary care medical center ED with approximately 65,000 adult ED visits annually. This study was reviewed and approved by the local institutional review board.

### Patient Population

We included adult (age ≥18 years) patients who presented to the ED with a chief complaint of abdominal, flank, or pelvic pain; had a normal mental status (defined as an initial Glasgow Coma Scale of 15); had a race and ethnicity of NH White, NH Black, Asian, or Hispanic/Latinx. Patients of all other races and ethnicities and those with missing race data were excluded. We also excluded patients who were not evaluated by an emergency physician (defined as an ED disposition of triaged in error, left without being seen, or straight to labor and delivery); those who eloped or left against medical advice within one hour of being roomed; and those with acute psychiatric emergencies (see Figure S1). If a patient had multiple qualifying ED encounters during the study period, we included only their first encounter.

**Population Health Research Capsule**

What do we already know about this issue?

*Racial disparities in pain management have been reported across the healthcare continuum, from the prehospital arena to the perioperative setting, including the Emergency Department.*

What was the research question?

*Do previously demonstrated disparities in analgesia extend to the contemporary adult ED population with acute abdominal pain?*

What was the major finding of the study?

*When presenting to the ED with abdominal pain, non-white patients are 27 – 38% less likely to receive opioid analgesia than white patients.*

How does this improve population health?

*Racial disparities in analgesia are pervasive and persistent. It must be a research priority to develop and validate interventions aimed at achieving racial and ethnic health equity.*

### Data Collection

Demographic and clinical data were directly exported from the electronic health record (EHR) by a trained data analyst. Data included age, sex, race and ethnicity (self-reported), Emergency Severity Index (ESI), initial pain score, a record of ED encounters within the prior 30 days for abdominal pain, opioid analgesia administration, non-opioid analgesia administration, ED imaging, pregnancy status, ED disposition, and surgery within 24 hours of ED arrival. The ESI is a score from 1 to 5 based on acuity and resource needs.^18^ If nursing documentation indicated that the patient denied pain and a pain score was not documented, a score of 0 was assigned. A random subset of 60 records were manually reviewed by the research team (AJ, BM, AW) to refine and validate the accuracy of all data fields. We did not find any errors in the extracted data. No data were manually abstracted.

### Definition of Variables

We followed guidelines from the US Centers for Disease Control and Prevention to classify patients’ self-identified race and ethnicity into the following categories: Non-Hispanic White (“White”), Non-Hispanic Black (“Black”), Non-Hispanic Asian (“Asian”), or Hispanic/Latinx.^19^,^20^ Opioid analgesia was defined as any medication that contained an opioid alone or in combination. Non-opioid analgesia was defined as any medication with an approved indication for pain without any opioid component. For both opioid and non-opioid analgesia, all routes of administration were included. Table S2 contains the list of analgesics included in the study. Imaging in the ED included plain radiograph, ultrasound, magnetic resonance imaging, and computed tomography of the abdomen or pelvis.

### Outcomes

The primary study outcome was the administration of any opioid analgesia during the ED visit. Secondary outcomes included the administration of non-opioid analgesia and any analgesia. We conducted subanalyses among non-pregnant patients, patients who underwent ED imaging, those who were admitted to the hospital, and those who underwent surgery within 24 hours of arrival to the ED. Patients were included in the admission cohort if they were admitted to inpatient or observation status.

### Data Analysis

We used descriptive statistics to characterize the study population. We then estimated the unadjusted and covariate-adjusted relationships between administration of opioid analgesia, non-opioid analgesia, and any analgesia with patient race/ethnicity using logistic regression (White race as the referent). The multivariable model included age, sex, ESI, initial pain score, and any ED encounter in the preceding 30 days. We selected these independent variables a priori based on published literature.^15^ Separate models were conducted for each subgroup described above. We performed analyses in Stata version 15.1 (StataCorp, LLC, College Station, TX).^21^ Statistical significance was assessed with α = 0.05 (2-sided). We did not adjust for multiple comparisons.

## RESULTS

### Patient Characteristics

A total of 13,841 encounters were considered for inclusion. Of these, 7,367 unique patients met inclusion criteria (Figure S1). A plurality were White (n = 3,314, 45.0%); 28.4% were Hispanic/Latinx (n = 2,092), 18.8% were Black (n = 1,384), and 7.8% were Asian (n = 577). Over half (n = 4,194, 56.9%) were female, and the median age was 47 years (25^th^–75^th^, 32–62 years). During the study period, 3,207 (43.5%) patients received an opioid during their ED encounter; 3,611 (49.0%) received non-opioid analgesia; and 5,095 (69.2%) received any analgesia (Table 1).

### Relationship Between Race/ethnicity and Analgesia Administration

In multivariable logistic regression models, non-White patients were less likely than White patients to receive opioids: odds ratio (OR) 0.73, 95% confidence interval (CI) 0.65–0.83 for Hispanic/Latinx patients; OR 0.62, 95% CI 0.54–0.72 for Black patients; and OR 0.64, 95% CI 0.52–0.78 for Asian patients (Table 2). Black (OR 0.73, 95% CI 0.62–0.85) and Hispanic/Latinx (OR 0.86, 95% CI 0.75–0.99) patients were less likely to receive any analgesia, but Black patients were the only group to receive non-opioid analgesia less often than White patients (OR 0.85, 95% CI 0.74–0.97).

### Subgroup Analyses

Results were similar across subgroups. The association was attenuated, however, among patients who underwent surgery within 24 hours of ED arrival (Table 2). Within this subgroup only Hispanic/Latinx patients were less likely to receive opioid analgesia.

## DISCUSSION

Our study found persistent racial/ethnic disparities in the administration of opioid analgesia to ED patients with acute abdominal pain. This finding aligns with a body of work documenting oligoanalgesia for patients of color in a number of painful conditions, including long-bone fracture and trauma.^15^ It also confirms and expands the prior work of Mills et al, despite differences in cohort composition with regard to race (45% White in our study vs 23% White in Mills et al), which found that non-White patients were less likely to receive any analgesia and waited longer to receive it. Their study included patients with abdominal pain or back pain but did not differentiate between non-White races. Our study expands this work by showing that patients who identify as Asian and Hispanic/LatinX are also vulnerable to disparities in analgesia administration.^16^ Our results also align with those from a cohort with acute abdominal pain from the National Hospital Ambulatory Care Medical Care Survey in which Black, Hispanic, and “other” race/ethnicities had lower adjusted odds of opioid analgesia receipt.^17^ Our study is the first to find that Black patients were less likely to receive non-opioid analgesia specifically, as well as opioid analgesia, suggesting particular disparities in undertreated pain in this group.

Prior studies did not differentiate non-opioid analgesia; our study found no difference in non-opioid analgesia for Hispanic/Latinx and Asian patients relative to White patients, but lower odds of non-opioid analgesia for Black relative to White patients. While this may reflect specific anti-Black biases that are not applied to other minoritized groups, this disparity was not seen in other subanalyses, for example among patients who underwent imaging studies. Importantly, though, we found disparities in type of analgesia provided, with non-White patients being less likely to receive the recommended opioid analgesia for acute abdominal pain, and pervasive disparities in all types of analgesia administration for Black patients. While our site has a robust diversity, equity, and inclusion curriculum for trainees, faculty, and staff intended to eliminate disparities in care, the results of the current study suggest that additional interventions are needed.

While there is a dearth of literature on interventions to address race-based disparities in care, the use of standardized and evidence-based protocols may reduce clinician biases and improve care for minoritized groups. In one study, a checklist that standardized care for ST-segment elevation myocardial infarction (STEMI) resolved gender-based disparities in time to cardiac catheterization and administration of goal-directed medical therapy.^22^ A recent study found that adherence to evidence-based guidelines (eg, stroke, STEMI, sepsis) did not vary by patient race/ethnicity and sex. We posit that establishing monitored criteria may be a useful tool in reducing race-based health disparities and minimizing the impact of implicit bias.^23^ Leaders in emergency medicine have recommended the development of specialized quality metrics that specifically include measures of health disparities.^24^

## LIMITATIONS

Our study’s primary limitations are those inherent to its retrospective design and the inability to control for unmeasured confounders retrospectively. However, we took multiple measures to mitigate potential confounding variables. While we expanded race/ethnicity categories beyond prior studies, we limited to four categories and, thus, were unable to account for heterogeneity within groups; patients of mixed race were also excluded but represented fewer than 10% of eligible patients. Our findings may have limited generalizability, as this was a single-center study. However, they are consistent with prior studies, including those from national datasets, suggesting these disparities are widespread and not due to local practice patterns.

## CONCLUSION

Asian, Black, and Hispanic/Latinx patients were significantly less likely to receive opioid analgesia for their acute abdominal pain compared with White patients, even when controlled for potential confounders. Black patients were also less likely to receive non-opioid analgesia. Black and Hispanic/Latinx patients were less likely to receive any analgesia. To eliminate disparities in analgesia administration, emergency medicine must prioritize research into the impact of interventions to mitigate race/ethnicity-based inequities in emergency analgesia.

## Supplementary Information





## Figures and Tables

**Table 1:** Patient characteristics: emergency department index encounter.^a^

Characteristic	Patients, N (%) (N = 7,367)
Race/Ethnicity	
Asian	577 (7.8)
Black	1,384 (18.8)
Hispanic/Latinx	2,092 (28.4)
White	3,314 (45.0)
Sex	
Female	4,194 (56.9)
Male	3,172 (43.1)
Nonbinary	1 (0.01)
Age, years^b^	47 (32–62)
Acuity level (Emergency Severity Index)	
1 - Resuscitation	19 (0.3)
2 – Crisis	2,414 (32.8)
3 - Emergent	4,761 (64.6)
4 – Urgent	172 (2.3)
5 - Non-urgent	1 (0.01)
First pain score^b^,^c^	8 (6–9)
ED encounter in prior 30 days	150 (2.0)
Opioid analgesia administered	3,207 (43.5)
Non-opioid analgesia administered	3,611 (49.0)
Any analgesia administered	5,095 (69.2)
Time to first opioid analgesia administered, hours^b^,^d^	2.8 (1.5–5.2)
Time to first non-opioid analgesia administered, hours^b^,^d^	3.6 (1.9–6.3)
Time to first any analgesia administered, hours^b^,^d^	2.7 (1.5–4.8)
Pregnant	71 (1.0)
Underwent any imaging study	5,004 (67.9)
Admitted to the hospital	2,133 (29.0)
Surgery within 24 hours of ED arrival	362 (4.9)

*ED*, emergency department.

a First qualifying encounter per patient during the study period.

b Data presented as median (25th–75th percentile).

c 342 patients missing pain score.

d Among patients administered that type of analgesia.

**Table 2:** Unadjusted and adjusted association of patient race/ethnicity and analgesia administration with subgroup analyses.

	Opioid		Any analgesia		Non-opioid analgesia	
		
	OR (95% CI)	aOR* (95% CI)	OR (95% CI)	aOR* (95% CI)	OR (95% CI)	aOR* (95% CI)
Race/Ethnicity						
Full sample (N = 7,367^a^)						
Asian	0.77 (0.64, 0.92)	0.64 (0.52, 0.78)	1.10 (0.91, 1.34)	0.97 (0.78, 1.21)	1.11 (0.93, 1.33)	1.05 (0.87, 1.26)
Black	0.79 (0.70, 0.90)	0.62 (0.54, 0.72)	0.97 (0.85, 1.11)	0.73 (0.62, 0.85)	1.02 (0.90, 1.16)	0.85 (0.74, 0.97)
Hispanic/Latinx	0.89 (0.80, 1.00)	0.73 (0.65, 0.83)	1.15 (1.02, 1.29)	0.86 (0.75, 0.99)	1.26 (1.13, 1.40)	1.03 (0.92, 1.16)
White	Ref.	Ref.	Ref.	Ref.	Ref.	Ref.
Non-pregnant patients (n = 7,296^a^)						
Asian	0.78 (0.65, 0.93)	0.64 (0.53, 0.78)	1.10 (0.90, 1.33)	0.96 (0.77, 1.19)	1.10 (0.92, 1.32)	1.04 (0.86, 1.25)
Black	0.80 (0.71, 0.91)	0.63 (0.54, 0.72)	1.00 (0.87, 1.14)	0.74 (0.63, 0.86)	1.04 (0.91, 1.17)	0.85 (0.74, 0.98)
Hispanic/Latino	0.90 (0.81, 1.01)	0.74 (0.65, 0.83)	1.16 (1.03, 1.31)	0.87 (0.76, 1.00)	1.26 (1.13, 1.40)	1.03 (0.92, 1.16)
White	Ref.	Ref.	Ref.	Ref.	Ref.	Ref.
Patients who underwent any imaging study (n = 5,004^a^)						
Asian	0.78 (0.63, 0.96)	0.64 (0.51, 0.80)	1.02 (0.80, 1.30)	0.86 (0.66, 1.13)	1.03 (0.83, 1.26)	0.98 (0.79, 1.21)
Black	0.99 (0.84, 1.17)	0.69 (0.58, 0.83)	1.39 (1.14, 1.71)	0.93 (0.74, 1.16)	1.20 (1.02, 1.41)	0.98 (0.83, 1.16)
Hispanic/Latinx	0.95 (0.83, 1.08)	0.69 (0.60, 0.80)	1.29 (1.10, 1.51)	0.86 (0.72, 1.03)	1.33 (1.17, 1.52)	1.09 (0.95, 1.26)
White	Ref.	Ref.	Ref.	Ref.	Ref.	Ref.
Patients admitted to the hospital (n = 2,133^a^)						
Asian	0.73 (0.53, 1.00)	0.64 (0.45, 0.92)	0.84 (0.58, 1.23)	0.71 (0.46, 1.08)	0.69 (0.50, 0.94)	0.63 (0.46, 0.87)
Black	1.19 (0.90, 1.57)	0.71 (0.52, 0.97)	1.12 (0.80, 1.56)	0.63 (0.43, 0.92)	0.92 (0.71, 1.19)	0.75 (0.58, 0.99)
Hispanic/Latino	1.28 (1.03, 1.60)	0.76 (0.59, 0.99)	1.17 (0.89, 1.53)	0.65 (0.47, 0.89)	1.08 (0.89, 1.33)	0.91 (0.73, 1.13)
White	Ref.	Ref.	Ref.	Ref.	Ref.	Ref.
Patients who underwent surgery within 24 hours of ED arrival (n = 362^a^)						
Asian	1.02 (0.46, 2.27)	0.92 (0.36, 2.04)	1.13 (0.41, 3.17)	0.92 (0.30, 2.83)	0.74 (0.36, 1.52)	0.70 (0.34, 1.46)
Black	1.18 (0.54, 2.58)	0.55 (0.22, 1.40)	1.65 (0.54, 4.99)	0.89 (0.25, 3.13)	0.92 (0.46, 1.82)	0.79 (0.38, 1.61)
Hispanic/Latino	0.94 (0.55, 1.59)	0.48 (0.25, 0.95)	1.19 (0.60, 2.38)	0.57 (0.25, 1.31)	1.10 (0.68, 1.79)	0.90 (0.53, 1.53)
White	Ref.	Ref.	Ref.	Ref.	Ref.	Ref.

*OR*, odds ratio; *aOR*, adjusted odds ratio; *CI*, confidence interval.

* Adjusted for age, sex, acuity, initial pain score, and any ED encounter in prior 30 days.
