# Supplementary material for: Racial Disparities in Opioid Analgesia Administration Among Adult Emergency Department Patients with Abdominal Pain
Source: West J Emerg Med. 2022 Oct 24;23(6):826–31. doi: 10.5811/westjem.2022.8.55750 (PMC9683779; doi:10.5811/westjem.2022.8.55750)
Supplement: Supplementary file 1 [file wjem-23-826-s001.pptx]

## Slide 1
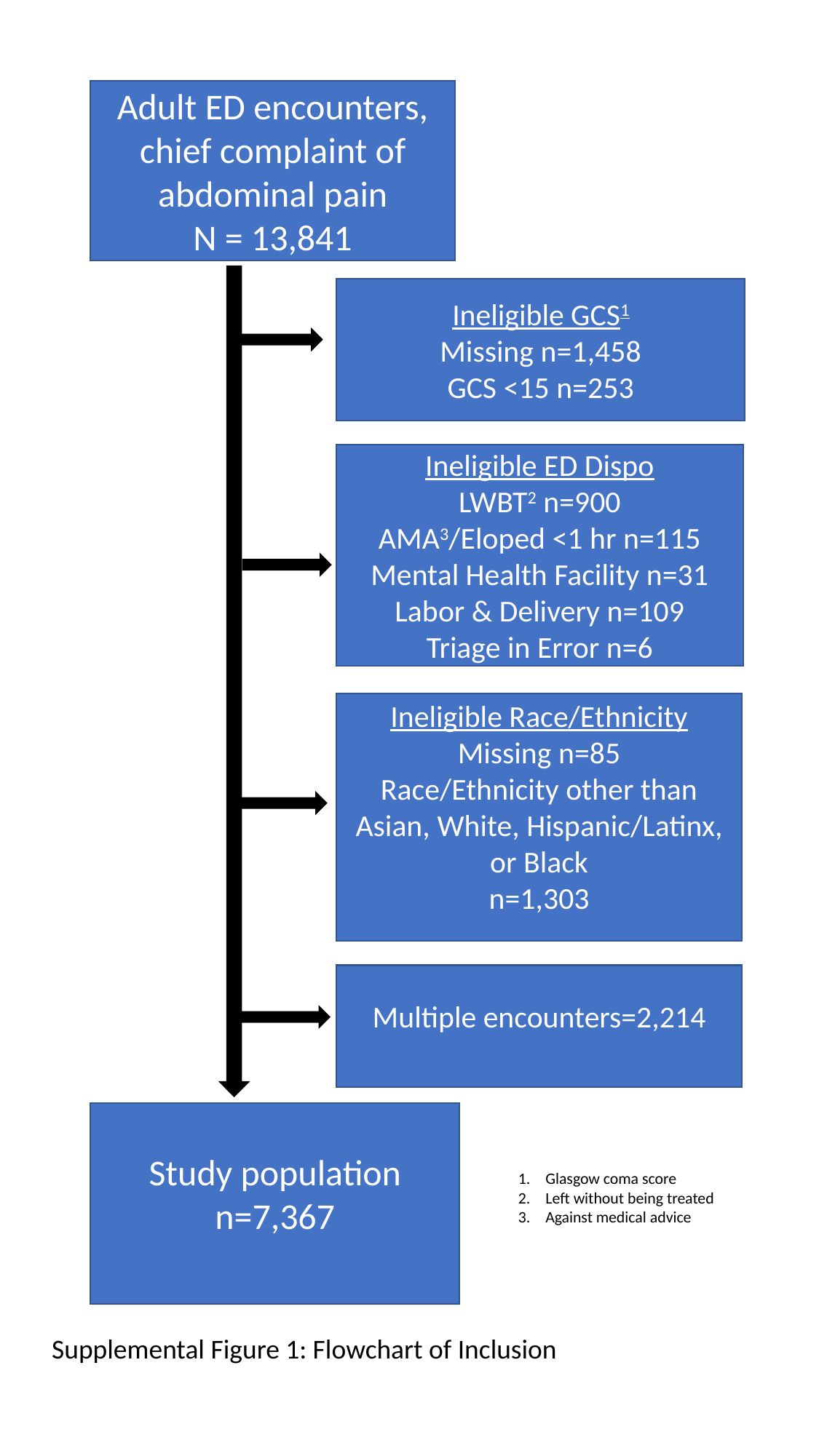

Adult ED encounters, chief complaint of abdominal pain
N = 13,841
Ineligible GCS1
Missing n=1,458
GCS <15 n=253
Ineligible ED Dispo
LWBT2 n=900
AMA3/Eloped <1 hr n=115
Mental Health Facility n=31
Labor & Delivery n=109
Triage in Error n=6
Ineligible Race/Ethnicity
Missing n=85
Race/Ethnicity other than Asian, White, Hispanic/Latinx, or Black
n=1,303
Multiple encounters=2,214
Study population
n=7,367
Glasgow coma score
Left without being treated
Against medical advice
Supplemental Figure 1: Flowchart of Inclusion
