# Supplementary material for: Racial Disparities in Opioid Analgesia Administration Among Adult Emergency Department Patients with Abdominal Pain
Source: West J Emerg Med. 2022 Oct 24;23(6):826–31. doi: 10.5811/westjem.2022.8.55750 (PMC9683779; doi:10.5811/westjem.2022.8.55750)
Supplement: Supplementary file 2 [file wjem-23-826-s002.docx]

**Supplemental Table 1: Included Analgesic Medications**

**Non-Opioid Analgesics**

| ACETAMINOPHEN |
| --- |
| ASPIRIN-ACETAMINOPHEN-CAFFEINE |
| BUTALBITAL-ACETAMINOPHEN-CAFFEINE |
| CELECOXIB |
| DICLOFENAC SODIUM |
| GABAPENTIN |
| IBUPROFEN |
| INDOMETHACIN |
| KETAMINE |
| KETOROLAC |
| LIDOCAINE |
| METHOCARBAMOL |
| NAPROXEN |
| MELOXICAM |
| PREGABALIN |
| MAALOX/LIDOCAINE |
| **Opioid Analgesics**  ACETAMINOPHEN-CODEINE |
| BELLADONNA ALKALOIDS-OPIUM |
| FENTANYL |
| FENTANYL-BUPIVACAINE |
| HYDROCODONE |
| HYDROMORPHONE |
| MEPERIDINE |
| METHADONE |
| MORPHINE |
| OPIUM TINCTURE |
| OXYCODONE |
|  |
| OXYCODONE-ACETAMINOPHEN |
| TRAMADOL |
